# Enhancing communication in oncology outpatient consultations: critical reflections from doctors

**DOI:** 10.5116/ijme.4ee2.0dc3

**Published:** 2011-12-16

**Authors:** Lynn Furber, Roger Murphy, Karen Cox, William Steward

**Affiliations:** 1 Department of Oncology, University Hospitals of Leicester, United Kingdom; 2 School of Education, The University of Nottingham, United Kingdom; 3 School of Nursing, The University of Nottingham, United Kingdom; 4 Department of Cancer Studies and Molecular Medicine, University of Leicester, United Kingdom

**Keywords:** Communication, reflection, continued professional development

## Abstract

**Objectives:**

The experiences of patients diagnosed with advanced incurable cancer and the doctors who conducted their medical consultations were studied in order to improve the understanding of what happens in consultations, when bad news is disclosed. The major objective of the study was to critically reflect upon doctor-patient communication, in such situations, with a view to considering future strategies for doctors’ continuing professional development.

**Methods:**

Sixteen patients and sixteen Oncologists, from a cancer centre in the UK were recruited into this ethnographic study. One hundred and fifteen episodes of data were collected from audio recorded consultations; interviews with doctors and patients and their relatives and observations of consultations. These data were analysed using a constant comparison method.

**Results:**

Interactions between doctors and patients are complex and consultations can be challenging for both of them. Some doctors spoke openly about their need for additional support to enhance their communication related competencies within Oncology consultations. These doctors wanted to observe their peers conducting consultations. They also wanted to receive feedback about their own clinical practices. These doctors stated that they wanted an open culture whereby they could talk freely about difficult and emotionally challenging consultations without fear of being considered incompetent by their Consultants, who act in a clinical supervisory role.

**Conclusions:**

To help practitioners consolidate their practice in such settings it is necessary to develop better collaborations among practitioners within clinical practice. Providing individual supervisory sessions or group workshops can facilitate reflective learning and provide an open and supportive learning culture.

## Introduction

While effective communication is important in any health care setting, it is essential in the cancer setting, due to the sensitivity of the information and the psychosocial impact a cancer diagnosis can have on patients. When imparting information to a patient, health care professionals need to consider and manage a number of emotions which are likely to be induced in their patients and themselves, throughout the course of the patient’s illness.^1^ In this context communication goes beyond basic skills. In the UK, the NHS Cancer Plan reported that communication skills training and development would become a feature of continued professional development.^2^ This commitment received further support through NICE guidance on supportive and palliative care which recommended that accredited courses should become available to help those working within cancer care.^3^ More recently, through the National Advanced Communication Skills Programme for senior health care professionals in cancer care (ASCT) there has been an initiative to combine a number of established communication skills training courses. The aim of the course is to improve the communication skills of senior health care professionals through experiential learning.^4^ Less experienced doctors working within oncology are excluded from this national advanced communications course. As such they may only receive communication skills training during pre-registration and continue to develop these skills as they engage in practice. This makes the focus upon doctors working within oncology especially interesting. People often learn from experience and we need to understand more about this and the issues people face if we are to help enhance practice in this area.Learning from experience is a natural form of learning, which is available to all, and helps facilitate personal growth and development through a combination of efforts.^5^ These efforts include, personal initiatives, educational support and peer support to help people discover what it is that they need to learn.^6^^-^^8^ Those who are not mindful of their practice and fail to reflect upon their actions and interactions with others may fall into the trap of believing they do not need to change and continue to engage in routine, standardised practices.^7^^,^^8^ Although experiential learning is considered to be a personal endeavour,^7^^,^^9^ it has been shown that benefits can result from those with greater expertise providing support and guidance to help facilitate this process.^7^^-^^11^ Connecting with others is an important aspect of developing ones learning, as explicit and implicit learning is more likely to take place through participation, working alongside others and sharing experiences to help overcome problems and consolidate learning.^10^ This is important, as disclosing bad news and talking about sensitive issues with patients in clinical practice is still identified as a difficult issue for doctors in particular ^12^^,^^13^ who are likely to question their competence and ability to engage in such discussions.^14^^-^^17^ Despite the emotional burden which exists within the context of cancer care it is rare for doctors to receive training and support to help them manage the emotional stress, which may be induced by difficult consultations with patients and their relatives.^18^ This phenomenon is of particular concern, when a good deal of a doctor’s time is spent communicating information and presenting treatment options to patients within a palliative or oncology context.^19^In order for practitioners to discuss and share sensitive information about their practice, trusting relationships need to be established between colleagues.^20^ If trusting relationships are not developed doctors are unlikely to disclose their concerns to colleagues in order to protect themselves from any experience of personal vulnerability.^20^ Furthermore, there is generally little scope for individuals working within the NHS to share their problems, experiences and knowledge with others or to coach each other through difficult experiences. This is partly a product of the ‘rational scientific paradigm,’ which dominates many clinical settings.^21^ Most clinical environments are not currently structured in such a way as to allow time for reflection and experiential learning and the sharing of knowledge and expertise.^22^^,^^23^This situation may be addressed in part through the provision of additional communication skills training for doctors within the context of real life clinical situations as they arise within clinical practice. Such an approach should not be based upon didactic learning styles but rather needs to involve, reflective practice and experiential learning within the clinical environment. However, before engaging in such approaches to learning and professional development, a greater understanding is needed about what goes on in consultations between doctors and patients and how they both experience imparting and dealing with difficult news, within highly complex social situations and social interactions. In this paper some of the challenges faced within clinical practice are reported. The context for the research was one where attempts were being made to manage emotionally challenging discussions with patients diagnosed with advanced cancer. Findings from this empirical research investigation indicated that more attention needs to be given to how doctors can be helped to develop their communication and interpersonal practices within this crucial area of medical care.

## Methods

### Study design and setting

Data for this study comes from a qualitative study conducted within a large cancer centre in an NHS Trust Hospital in the UK. An ethnographic approach was used to explore the experiences of patients diagnosed with advanced incurable cancer and the doctors, who conducted their medical consultations, particularly with regards to the way sensitive issues and the communication of bad news were dealt with. A longitudinal, prospective approach was applied, with a view to exploring doctor and patient experiences as they occurred within a series of medical consultations; from initial referral and as patients progressed through their illness. An interdisciplinary approach, drawing on sociology, health and education disciplines and knowledge bases was applied to this study. The intention was to reflect upon such data to explore how it might inform future attempts to improve doctors’ communications and interactions with their patients. Additionally, the study set out to consider ways in which future professional development activities in this area could be designed based upon a deeper understanding of the current experiences of a sample of doctors and patients.

### Recruitment

A purposive sampling method was applied to this study. Four consultant oncologists (those who treated patients with advanced cancer) and all the specialist registrars (SpR) working within an oncology department were asked to participate in this study. A SpR is a doctor who is in the process of receiving specialist training in oncology. Fourteen SpR’s worked within the oncology department at this time. All of the doctors were provided with written information about the study. Doctors who expressed an interest in participating in the study were given an opportunity to seek further clarification about the study. Written informed consent was then obtained from all the doctors who participated.The researcher liaised with the participating consultant’s clinic co-ordinators on a weekly basis to identify eligible patients. Patients were considered eligible if they had a new diagnosis of advanced incurable cancer or a recurrence of disease; had been given a diagnosis; had a median survival of between 6-12 months; and were over 18 years of age. A letter of invitation and study information sheet were sent to each patient in the post. In total 62 patients were invited to take part in this study. Those who expressed an interest were met by the researcher prior to their first consultation with the oncologist. The purpose of the study was explained to each patient again and patients and/or their companions were given an opportunity to ask questions. Those who agreed to participate provided written informed consent. Recruitment took place over a 9 month period.

### Data Collection

Over a period of 20 months, 115 episodes of data were obtained through multiple methods of data collection. Prior to conducting the main part of the study, Consultants and SpR’s were asked to describe, during interviews, how they interacted with patients, how they developed their communication skills and how they felt they communicated bad news to patients diagnosed with advanced incurable cancer. These interviews were conducted to obtain a broad, generalised understanding about how each doctor perceived their communication skills and interactions with patients, and what aspects of the consultation experience they found to be challenging or most fulfilling. These interviews were audio recorded and transcribed verbatim. In the main part of the study consultations between doctors and patients were both observed and audio recorded to provide a record of the conversations which took place. Whilst conducting these observations the researcher deliberately sat on the periphery of the consulting room, in a position which enabled her to observe what occurred in the course of the consultation. Field notes were recorded after every consultation.A key feature of the study was an exploration of the combined perspectives of doctors and patients. This was used to develop a broader understanding of what they each believed transpired during their interactions with each other. It was envisaged that their separate accounts would provide a richer and more inclusive insight into their experiences. This is one of the features that set this study apart from previous studies in this area. All the patient interviews were conducted within 1-5 days of the consultation, either within a private room situated within the oncology department or in the patient’s home.

### Participants

The response rate for doctors was 89%. Sixteen Oncologists participated in the study; 3 Consultants and 13 SpR’s. Once recruited, all of the doctors remained in the study. All of the doctors participating had received some form of communication skills training; either as a medical student or post registration. Six doctors were interviewed and observed on only one occasion; two doctors on two occasions; one doctor on three occasions and two doctors on six occasions. The number of interviews and observations conducted with each doctor was dependent upon the number of patients from the study sample who happened to attend one of their consultations.This was dependent upon whether or not they were present in clinic when the patient attended their consultation. The mean number of consultations per doctor was 2.27. Table 1 shows the doctor characteristics.

**Table 1:** Characteristics of doctors (N=16)

Gender	Consultant	SpR
Male	2	6
Female	1	7
Total	3	13

The response rate for patients was 26%. In total 16 patients agreed to participate in the study; 3 patients had experienced a recurrence of their disease, having initially received surgical intervention within the past 18-24 months. The remaining 13 patients had been newly diagnosed with cancer within the past month. The main reason given by patients for not participating in the study was that they didn’t feel well enough to participate in research. Table 2 shows the patient characteristics. During the study, 10 patients were lost to follow up, which means they were only interviewed once; 6 patients were seen again for a second consultation and 3 patients were seen again for a third consultation. The mean number of consultations per patient was 1.56. The names of all research participants were replaced with pseudonyms to ensure their anonymity.

**Table 2:** Characteristics of Patients (N=16)

Variable	N
Gender	
Male	12
Female	4
Age	
<59	5
>60	11
Marital Status:	
Married	15
Single	1
Cancer Site	
SCLC	1
Gastric	2
Oesophageal	4
Pancreatic	6
Other	3
Ethnicity	
White British	15
Asian	1

### Analysis

The constant comparison method were used to help identify, define and refine the theoretical categories as they emerged from the data.^24^ The following analysis techniques were adhered to: Coding; memo writing; axial coding and theoretical sampling.^24^ Transcripts and field notes were imported into NVivo 7 software to assist in the coding, management and analysis of data. Questions (how, what, when, why, where) were asked about a whole range of issues including; the significance of observations, interpretations of experiences, meanings behind comments and about the characteristics of concepts. The coding format was informed by the theoretical framework of awareness context. Awareness context theory was first developed in the 1960’s following an investigation to explore the awareness of dying in American hospitals.^25^ In brief, awareness context theory considers how people manage and share information with each other, particularly when the information is of a sensitive and life threatening nature and how emotions influence cognitive understanding and subsequent interactions.^25^^-^^27^ The theoretical framework and research objectives helped the researcher interrogate and order the data to consider, for example, how meaning was attributed to experience, how information was managed, and what helped or hindered the way people communicated and interacted with each other.

An iterative process of data collection and analysis was utilised with each stage informing the other. As themes and concepts emerged from the observations, interviews and recordings of consultations it was necessary and appropriate to pursue these further in subsequent interviews and observations. Constantly making comparisons with the data helped us analyse the data from interviews, observations and recordings of consultations, in order to describe and interpret the views of those studied and bring substantive meaning to their experiences. This approach allowed the research to consider variations within the data and act upon them accordingly to retain a sense of focus.^24^ In addition, respondents who were seen on more than one occasion were provided with a summary of any key points they made to seek clarification or to encourage further elaboration.

### Ethical Approval

Approval for the study was obtained from the Leicestershire, Northamptonshire and Rutland Local Research Ethics Committee (LREC) and Research and Development Department.

## Results

Interactions between doctors and patients are complex and challenging. The results presented in this paper focus on two themes which emerged from the data. The first theme relates to ‘Managing emotion in medical consultations’. This theme highlights some of the challenges doctors’ face in clinical practice, particularly in regards to addressing the emotional needs of patients diagnosed with advanced cancer. The second theme, ‘A need for additional support within clinical practice’ demonstrates how doctors want to develop their communication skills further. These themes emerged out of the data and can be used to consider future strategies for doctors’ continued professional development. The full outcomes of the study^11^ show that, while some doctors were not always unreceptive to the information needs of their patients, they were also not always mindful of the part patients played within the consultation, in terms of emotional cognition and work. Names of patients and doctors have been changed and will be identified by their status of patient, doctor or relative, their gender and participant number.

### Managing emotion in medical consultations

Despite the certainty that most people will be upset having been told that they or a loved one has a life threatening illness, there was a tendency for doctors, patients and their relatives to express or show some difficulty in sharing these emotions with each other and their colleagues. This phenomenon is complex, but having observed a number of consultations, several factors arose which were of relevance. These included whether or not the doctor felt that they had the relevant skills to show empathy to their patients and how they sought to learn appropriate interactions. For some doctors though, they were not convinced it was part of their role to explore emotional distress or offer emotional support.The need to offer emotional support and empathy to patients was important for a number of doctors in this study. They wanted to find suitable ways of expressing empathy which they hoped would convey a message that they were genuinely compassionate to the patient’s situation and that they wanted to help them, but they did not always know how to demonstrate this appropriately. Some doctors were however, more insightful of their need to improve their skills than others. For example, DrM1, realised he had difficulty offering emotional support through the use of ‘touch’ and by this we refer to the touch of an arm or a knee to make some form of physical connection with a patient who may be distressed. It was believed that using ‘touch’ in this way would help demonstrate empathetic understanding. DrM1, recollected touching the knee of a male patient, having disclosed some bad news, but the patient moved away from him and he felt ‘it had actually made things worse and I thought oh damn’ (DrM1). He believed that the patient was not receptive to the use of touch and as such his intention to offer empathy failed as his interaction was rejected by the patient. DrM1 became wary of using touch in future interactions but was worried that he might appear ‘uncaring’ or ‘standoffish’ but didn’t know what other techniques he could use.For the majority of the SpR’s, learning how to offer emotional support and developing their own styles of communication appeared to be a lonely journey and one of trial and error. One bad experience was enough to deter them from using a particular style of communication. For example, DrF1 felt that she needed to be less honest with patients when disclosing a poor prognostic outlook to patients after she had disclosed information to a patient who was not willing to hear what she had to say. As DrF1 explains;

*‘Well it was one of those things. At the time it seemed the right things to do, but looking back, now I know how they reacted I would have just said well let’s see how you are in a year and I would have left it at that. But you know hindsight is a wonderful thing. Knowing what I know now I wouldn’t have forced that information on them which is effectively what I did.’* (DrF1)

DrF1, felt she had been criticised for the way she had handled this consultation by her senior doctor. In this case, PtM1 refused to see DrF1 again in clinic and described how the doctor ‘had no right to talk to me like that’ (PtM1). DrF1 went on to explain that she would be cautious in the way she communicated prognostic information to patients in the future and would not be guided by her instinct to be truthful in such an open and blunt style. In addition she appeared genuinely upset by the way PtM1 had responded to her following the consultation.Some of the more experienced doctors mentioned ways in which they felt their interactions had changed over the years. Some felt that as they had grown in experience, their confidence had developed and they felt better able to judge how to respond to patients and their relatives in any given situation. This is brought to life in the following example:

*‘I think I have become more able to, I may be wrong, but my interpretation is maybe I can understand a bit more about what they are feeling more quickly and I have probably seen most of it on several different occasions and can now find the words more easily than I used to.’* (DrM2)

Yet, even more experienced doctors came across situations, within consultations, which they found difficult to manage. For example, DrM2 was faced with a patient’s husband, who appeared to be angry and upset during a consultation, but would not disclose what he was thinking. During this consultation, DrM2 thought the husband of his patient was quite angry as he resisted attempts to engage in the conversation and DrM2 described feeling uncomfortable. As this part of the consultation unfolded, the atmosphere within the consulting room was tense and DrM2 appeared hesitant about how he should proceed. PtF2 came to the rescue, by breaking the silence and proceeded to explain that her husband was upset and moved the conversation forward in a light hearted manner and DrM2 followed her lead.For some doctors though, their interpretation or assessment of their personal attributes was different to that of their patients. Some doctors were concerned that they might become complacent about the way they interacted with people. Doctors who tended to be complacent failed to question the way they interacted with patients and their perception of self-awareness was often significantly different to the image they conveyed to patients. For example, one doctor described during his initial interview, how he felt he was ‘sensitive but less sensitive’ with patients in consultations than he used to be, because for him the consultation had become ‘routine’ and no longer a ‘unique experience’ (DrM3). Yet, he was observed to follow a very rigid structure, within his consultations, which prevented PtM3 from addressing his concerns, as and when he chose to express them, within the consultation. In this case, PtM3 didn’t mind that DrM3 structured the consultation in this way because he felt he ‘was disrupting the flow of the consultation and taking the conversation all over the place’ (PtM3). Yet, PtM3 did feel that DrM3 was not particularly sympathetic.DrM3 still hoped however, that he ‘managed to do a good job’ (DrM3). Yet, two patients were particularly distressed by this doctor’s lack of sensitivity and refused to be seen by him in clinic. In such cases, the patient was unlikely to tell the doctor how displeased they were for fear that doing so would compromise their care in some way. As one relative explained following a previous consultation;

*‘....you know it is not etiquette to say why didn’t you do this and why didn’t you do that......you don’t want to get their back up do you? You don’t want them to think oh he’s going to be a bloody nuisance. Is she going to get the same treatment or is she going to get nothing?* (RtM1)

In addition PtM4 who was distressed by the way DrM3 communicated with him explained, ‘you don’t mess on your own doorstep’ (PtM4). As such the doctor was unaware of the effect they were having on their patients and an opportunity to reflect and learn from their practice was lost. In the following example, the patient has been told that the treatment he was pinning his hopes on was no longer appropriate due to a deterioration in his health. While the couple accepted that DrM3 was telling them the truth, they were unhappy with the way the information was disclosed:

*‘He said there’s no good going back to him, he can’t do anything for you. So he said you have to take what life throws at you I’m afraid. And he spoke like that and it hurt. He was telling the truth, I don’t doubt that but it was the way he did it. It really upset us. We don’t want to complain about him, but he’s got to learn to be a bit more sensitive than he was.’* (RtF1)

On the occasion that emotions were publicly displayed or alluded to by the patient or their relative, some doctors failed to notice or react to the patient’s cues sensitively, whereby concern and empathy may have been demonstrated through their interactions. For example, one doctor was observed to direct her attention to the patient’s notes and computer screen during a consultation, whilst her patient tried repeatedly to tell her he was feeling depressed. The following extract is a good example of this:

*‘I feel as though I am wasting what time I have left because I can’t think to do anything particularly. I can’t be bothered. I am quite content just sitting there for hours on end really, thinking things over and obviously I get depressed from what I am thinking.’* (PtM2)

The doctor didn’t respond to this information and proceeded to ask PtM2 questions about his physical symptoms. When asked at a later date how she thought PtM2 was feeling DrF2 replied that she thought he was having ‘a normal reaction to his illness.’ While she acknowledged that the emotional needs of her patients were important, her primary role was;

‘As an Oncologist, what we are trying to do is control the disease and this is our primary role. I have to draw the line and say I can give some support but only to a point.’ (DrF2)

The point at which this support began and ended was unclear, as was the type of support DrF2 felt was required. It was not uncommon for doctors to focus on the medical-technical aspects of patient care and avoid emotionally charged situations. If DrF2 had questioned PtM2 about how he was feeling she would have learnt that he was scared of dying alone and in pain without the support of the medical profession. PtM2 simply needed reassurance that he would receive appropriate support when needed. In this case, PtM2 was not critical of the way DrF2 conducted his consultation, but it was evident that his concerns had not been alleviated during the consultation and he described a general concern that now he was no longer receiving active cancer treatment the medical profession were less interested in his wellbeing.Although PtM2 did take steps to tell DrF2 how he was feeling it was not uncommon for patients to present a jovial image within the consultation in an attempt to hide how they were really feeling. PtM2 had on previous occasions presented a jovial image because he didn’t ‘want to appear miserable in front of the doctor’ (PtM2). As PtM2 had explained how he tried to be jovial, despite how he was feeling, during his first interview, the researcher realised the significance of his demeanour in this second consultation, and subconsciously willed the doctor to listen to what he was telling her. On such occasions when patients did try to hide their true feelings, the relative was more likely to offer a truthful account of the patient’s emotional wellbeing, but again their views were infrequently sought, particularly if they became upset in the consultation. This was particularly so if the doctor felt they ‘needed to focus their attention on the patient’ (DrF3). In taking this stance, the opportunity to learn some vital information about the patient was lost. In addition, it was evident through their discourse that some doctors found it particularly difficult to deal with the emotional expressions of relatives, who appeared to them to be; ‘militant, protective or angry’ (DrF6). This is expressed in the following extract:

*‘It is always difficult to deal with angry relatives. I probably, honestly don’t deal with it that well, from the fact that it still affects me.’* (DrF4)

*‘If I am pre-warned that someone is angry I make sure I am absolutely 100% sure of what has gone on before they come in. It does make you feel anxious, not scared but makes you feel uncomfortable.’* (DrF5)

*‘I think it is very emotionally challenging, Oncology. A lot of what we do is palliative care but we do it in a setting where people hope or expect they will improve. Managing people’s expectations are very difficult, very time consuming and very draining on your psychological reserves.’* (DrM4)

Negative experiences often evoked a stronger emotional response on the part of these doctors that meant they were better able to recollect what had happened to them and were able to remember situations in more detail. As such difficult experiences within medical consultations tended to offer a greater learning opportunity.

#### A need for additional support from within clinical practice

Most of the doctors who participated in this study had received communication skills training as part of their medical education, and some had attended a communication skills course post registration, as part of their continued professional development. Two key elements of the training that doctors liked were being able to try new skills in a safe learning environment, and being able to develop self-awareness through peer assessment and feedback, particularly in the context of breaking bad news and disclosing sensitive information. This is reflected in the following quotes:

*‘.there are new strategies that you can try in a protective and safe environment to see how it feels and whether or not it works.’* (DrF3)

*‘We were videoed talking to a patient and they then went through our body language and mannerisms. That was really educational because you don’t often realise what you are doing.’* (DrM5)

It was however, difficult for some doctors to transfer or sustain some of the skills they learnt on these courses to real life clinical situations. For example, DrF3 wanted to develop better time keeping skills but found it difficult to apply the techniques recommended to practice. In addition, another doctor felt that the techniques he was given to try in practice proved unhelpful as he became confused about what techniques he should use in any given situation, and ‘rather than have a structure to pin it on, I was kind of going I can take this or that but it went horribly wrong’ (DrM1). As he became ‘muddled’ about how to demonstrate emotional support and empathy DrM1 spoke of needing the help and support of his Consultant to help him develop his communication skills further and offer guidance. In this case, the doctor felt the support offered was ‘superficial without intent’ (DrM1).It was however, rare for SpR’s to turn to their Consultants for support and guidance. There was a consensus of opinion that the SpR’s could speak to each other or their Consultants about medical-technical matters but they could not talk freely about their experiences of communicating bad news and the emotional issues that were associated with this because it was not embedded in the medical culture. This is expressed in the following quotes:

*‘ I don’t think there is openness at all. I think if you start saying I have struggled with this you start looking as if you are not doing your job very well. The hierarchy is difficult. You want to impress your consultant and I think deep down that is what most doctors want to do or show that you are coping. And to admit to someone, actually I don’t think I handled that very well. It is very hard to do that because you are sort of admitting a failure in some way and it is engraved into you that you don’t do that.’* (DrF7)

*‘....you go around the edges of how important it is when discussing it with colleagues, not necessarily show how it affected me. Yes there are certain people I will talk to but quite often it is my wife at home.’* (DrM6)

Developing an open and supportive culture in clinical practice to aid learning and professional development was prohibited through personal and cultural climates. Doctors generally chose to communicate their concerns about difficult and challenging consultations to people outside work and chose not to speak to their Consultants because they believed them to be superior and feared being judged unfairly or for fear of compromising their position. Such a restrictive culture was not perceived satisfactory to the learning needs of some of the doctors;

*‘This is a really bad thing but I don’t think we ever sit down and go right what did you do or rarely do you sit down and go what was good or bad about the consultation.’* (DrM1)

*‘I don’t think we are very good at providing clinical supervision. You need to bounce things off colleagues. Someone to acknowledge yes it is ok to be sad about that is all you need really.’* (DrF3)

Two key elements emerge out of these quotations. Firstly, some doctors want to talk to someone within the clinical environment to help them reflect upon consultation experiences. Secondly, some doctors want someone to offer support in terms of recognising that consultations where bad news is disclosed on a regular basis will have an emotional effect on the doctors personally.In addition, some doctors explained how they wanted to receive feedback about their clinical performance from real life consultations with patients. This is expressed in the following quotes:

*‘You don’t get any feedback about what you did well or what you could improve on or what someone else who is medically trained thought. Or just someone to say that was difficult. There is none of that and you know I think we would all like to know if we have done things well or badly or if we could improve on things. It would be helpful from time to time.’* (DrM6)

*‘There will be times when you want to learn from a situation but it is difficult to talk to someone because there is nobody there. That is something that needs addressing.’* (DrF7)

Interestingly it was not uncommon for the doctors to use our research interview with them as an opportunity to reflect on the consultation as it helped them think about what they had achieved in the consultation and what they might have done better. This was simply as a result of the researcher asking questions about the consultation experience. Several doctors welcomed the opportunity to meet with the researcher. Having someone sit in and observe them conduct consultations was also generally welcomed. In addition to receiving feedback, others valued the opportunity to observe their colleagues interacting with patients:

*‘Looking at how other people do it either in a very good or a very bad way has taught me what to do and what not to do, which was more helpful than going on a course.’* (DrM7)

*‘I think the best training I have had is observing other people. You see some people do it well and you see some people do it badly and you think I really don’t want to do it like that or I do want to do it like this.’* (DrM3)

Much as they would like the opportunity to do this, such opportunities were not all that frequent and appeared to be ad hoc opportunities. This was generally related to busy demands and constraints imposed on their jobs and a perceived lack of support from some of the Consultants.

## Discussion

Steps have been taken over the past fifty years or so to help health care professionals to improve the way they communicate and interact with their patients, and to improve the way in particular, doctors develop relationships with patients. While patients want their doctors to communicate and interact with them in a manner that shows respect, interest, empathy, compassion and truth, this does not always seem to be fulfilled in practice. In this study some doctors were preoccupied with meeting their own needs and objectives, in an allotted time, and failed to notice or be receptive to the individual patient’s emotional needs and their requirement for emotional support.^28^ In this event, patients often left the consultation with unmet needs. If a patient felt their needs had not been addressed within the consultation they were also likely to be dissatisfied with their consultation experience.On a number of occasions patients described aspects of their consultations, whereby they felt that the doctor had been insensitive. These were not always consultations, which had been observed as part of this study, but what transpired in one consultation had a knock on effect on how patients perceived future consultations. If a doctor had been insensitive in the way that they disclosed information or in the way that they attended to or disassociated themselves from the patient’s emotional wellbeing, the patient was unlikely to disclose their displeasure to the doctor. Some patients were fearful of disclosing their displeasure for fear of compromising their care. As such a lot was left unsaid and doctors were often oblivious to the impact that their interactions had on a patient. It may be that obtaining feedback from consenting patients about their experiences, and sharing this information with doctors, might provide a good learning opportunity, as lessons can be learnt from real life clinical situations. During some of the interviews with doctors, they used the opportunity to talk to the researcher, as a reflective exercise and as an opportunity to try and review their performance. In addition, some doctors wanted to know what the patients had said during their interviews and one doctor was particularly upset when the researcher was unwilling to disclose such information, because the patient hadn’t provided consent for her to do so. The doctor felt that a great learning opportunity had been missed.While some of the doctors appeared to reflect on their actions/interactions with patients and were fearful of becoming complacent in their practice, others appeared to be less receptive and some appeared to be in danger of becoming complacent. Why some doctors were more conscientious about learning from their practice than others was not fully clear from the findings of this study. It may be that some were more able to make a connection between how they felt and how they felt the patients and/or their relative many have perceived their interaction. Some of the less experienced doctors were more conscious and thoughtful about managing patient emotion within the consultation, but tended to learn through trial and error, which could have a negative impact on the patient. For example, when DrF1 disclosed information to PfM1 about his poor prognosis it was evident that she didn’t know how to manage the emotional reactions of the patient and his partner and the remainder of the consultation was compromised, as was her subsequent relationship with the patient. DrF1 felt that following this event she had been reprimanded for the way she had managed this consultation. Rather than reflecting further upon how she might have managed the emotions of PtM1 and his partner following the disclosure of his poor prognosis, she seemed instead to conclude that in future she should lie to patients who have a poor prognostic outlook. This case, was a serious example of what can happen when doctors do not know how to handle patients’ (or their own) emotional states and when there is a lack of clinical support and supervision.Other studies have shown that while some doctors describe themselves as competent and confident, in responding to the emotional expressions of patients, they rarely demonstrated this behaviour in their consultations.^29^ This was despite having confidence in their ability and prior training in communication skills. Some of the more experienced doctors in this study felt they had learned to interpret emotions more easily but did not always express this certainty through their interactions. On occasion they were observed to be uncertain about how they should respond to the patient or relative who appeared to be upset. In some cases, the doctor simply chose to distance themselves from any expression of emotion and chose to close down further emotional disclosure. In doing so, they may have failed to learn some important information about the patient’s wellbeing. This is despite a move towards a collaborative approach to health care and patient interaction, whereby the emotional needs and concerns of patients are to be considered and addressed.^30^This is clearly a problem, which may have a negative impact on patient care and satisfaction. It is inevitable that the information disclosed to patients in an oncology setting is going to induce a number of emotional reactions and doctors need to be competent at attending to these emotions, within the context of the medical consultation. Doctors need to attend to the emotional needs of patients by asking them how they are feeling or by giving the patient an invitation to describe their emotions by recognising that this information must be difficult to hear. When doing so doctors need to be mindful that on initial probing, patients may often deceive them about their true feelings. In this context it may be appropriate to offer doctors who have become complacent or regimented in the way they communicate with their patients, some form of ‘awareness’ training.An achievable recommendation, appropriate for UK health care practice would be to help doctors and other health care professionals consolidate their communication skills learning. Whilst attending communication skills training courses is important, there is a need to develop a more sustained communication skills learning environment within clinical practice. Doctors need to learn communication skills and test out their skills within a safe learning environment, but then they need help to apply these skills within clinical practice and to reflect on their endeavours. Specifically, some doctors have explained how they would like someone within the clinical environment to help them reflect upon consultation experiences. Some doctors also feel it would be helpful if there was someone to offer them support in terms of recognising that disclosing bad news to patients on a regular basis will have an emotional impact on them.Doctors participating in this study have clear views about how to implement training models within the clinical environment which could be borne in mind when considering policy/organisational related recommendations. For example, it was deemed important to sit in and observe colleagues conduct consultations and to have others sit in and observe their consultations with subsequent opportunity for providing feedback. It has been suggested that expert peers can help even experienced doctors consider their values, beliefs and current practices^31^ and help them consider the ramifications of their actions/interactions during medical encounters. In addition, peers may be able to help them consider alternative approaches and techniques in practice until they are able to internalise the knowledge or skill into their existing practice,^32^ an exercise which has been considered valuable in other professional groups.^33^ Doctors participating in this study felt that it was necessary to learn from other members of the medical team to help develop and inform their clinical practice. Connecting with other members of one’s team has been regarded as a desirable approach to learning.^10^ Yet, doctors in this study were rarely, if ever afforded the opportunity to ‘connect with others’ in relation to developing their communication skills. The role of other people in supporting the learning needs of doctors is one that requires further attention. A review of the literature, pertaining to experiential learning has shown how the role of others can help encourage and support practitioners to develop their skills, confidence and competence within clinical practice.^7^^,^^10^Furthermore, some of the doctors felt they were unable to share their concerns with colleagues because the medical culture tacitly prohibits such disclosure, for fear of being judged incompetent. Interestingly, several doctors described how they would like to talk to someone who has clinical experience, but is not one of their Consultants. Only one Consultant described how they spoke to a colleague from within a different discipline of medicine to talk about difficult consultation experiences. This apparent tendency to bottle up poor consultation experiences has implications for practice and openness between professionals. Learning from difficult challenges and ‘hard knocks’ in the workplace can be seen to present an exciting learning opportunity^29^ and many of the doctors valued the importance and benefit of learning through an exposure to real life situations. Implementing clinical supervision might well fulfil some of the learning needs of doctors working within this specialty.If left unchallenged and unsupported people may feel too overwhelmed and their motivation to explore various strategies within their practice may be ‘stunted’.^32^ These problems have been echoed elsewhere in the literature^17^ where it has been suggested that the extent to which professionals are prepared to disclose their practice requires further investigation. Despite the fact that most of the doctors valued the importance and benefit of experiential learning within their work environment, the learning culture within medicine does not currently fulfil this need sufficiently. In order to explore this further it has been suggested that an examination of the ‘micropolitical discourse’ which resonates throughout the medical profession is required to understand how learning may be affected.^34^

## Conclusions

The findings of this study clearly indicate that shortcomings in doctor-patient communication occurred in the contexts studied. To help doctors consolidate and build upon their communication skills it may be necessary to develop better collaborations among doctors, within clinical practice. This is irrespective of their years of experience or perceived level of experience. Providing group workshops and / or individual supervisory sessions may be more likely to facilitate reflective and experiential learning and provide an open and supportive learning culture.It is also crucial that doctors receive support and guidance to help them address the complex nature of emotional care both within their profession – learning to support each other – to transcend this philosophy into patient care. From this perspective, it has been argued that by meeting the learning needs of doctors within clinical practice through experiential learning in this setting and context of care will influence the delivery of care for patients in the future. This is particularly relevant as patients have been shown to be influential in terms of how the consultation may proceed. To this end, doctors need to be mindful of the part patients play within the consultation and receive ‘awareness’ training.

### Strengths and limitations of the study

An opportunistic approach allowed us to collect very rich data from a sample of patients diagnosed with an advanced incurable cancer and doctors, who consulted with them about their illness. This allowed us to access some startlingly contrasting perspectives, which highlight we argue some very unfortunate clashes between the needs of these patients and their relatives and some of the consulting strategies employed by a small sample of oncology doctors. In this context it was however unfortunate that some patients were only interviewed on one occasion because they were either too unwell to continue with the study or their care was transferred to a different Oncologist who was not participating in this study. This was always going to be a problem though, because of this particular patient group. Secondly, a decision had been made early on to attend patient consultations, where bad news was likely to be disclosed, for example, when a patient’s treatment was to be discontinued, or when they attended a consultation, to hear the results of clinical investigations. Yet, patients spoke of difficult consultations which had not been observed and which had a negative impact on subsequent consultations or perceptions of doctors. As a consequence the doctor’s perspective of the consultation was missed. A key strength of the study centred on the research being able to capture experiences from patients diagnosed with an advanced incurable cancer and their doctors, thus enabling a comparison of a combination of perspectives throughout the patient’s illness. Thus what we have is some highly valuable data collected from an opportunity sample of doctors and patients. This has been collected using a highly ethical approach in a study that was conducted in a very sensitive area of clinical practice.

## 

### Conflict of Interest

The authors declare that they have no conflict of interest.
